# Supplementary material for: Breeding seasonality of Tylopoda: Expected patterns in Old World camelids but an exceptional pattern in South American camelids
Source: PLoS One. 2025 Dec 22;20(12):e0339382. doi: 10.1371/journal.pone.0339382 (PMC12721552; doi:10.1371/journal.pone.0339382)
Supplement: S1 File — (DOCX) [file pone.0339382.s002.docx]

SUPPORTING INFORMATION

**S1 Table. Mean gestation period in days of camelid species.**

| **camelid species** | **gestation period (days)** | **source** |
| --- | --- | --- |
| *Camelus bactrianus* | 374-419 402 | (1) (2) |
| *Camelus dromedarius* | 385-410 | (1) |
| *Lama glama* | 335-350  348-368 | (1)  (3) |
| *Lama guanicoe* | 345-360  346 | (3)  (4) |
| *Vicugna pacos* | 335-350  343  345 | (1)  (5)  (6) |
| *Vicugna vicugna* | 330-350 | (3) |

**S2 Table. Duration of lactation in camelid species.**

| **Vicuna** | | |
| --- | --- | --- |
| 6-8 months |  | (7) |
| Until the next young is born |  | (8) |
| **Alpaca** | | |
| 4-6 months | Time of weaning | (9) |
| At least 6 months |  | (10) |
| **Guanaco** | | |
| 7 months | Restart of nursing when the new young is born, final weaning with 13-15 months of age | (11) |
| 8 months |  | (12) |
| 11-15 months | Joint nursing of the yearling and the newborn | (7) |
| At least a year | First decrease of lactation after 4 months | (13) |
| **Llama** | | |
| 4-6 months | Time of weaning | (9) |
| 6-7 months | Time of weaning | (14) |
| 6-7 months |  | (15)  (16) (14) |
| **Bactrian camel** | | |
| 10-12 months |  | (17) |
| 14-16 months |  | (18) |
| 16 months |  | (19) |
| **Dromedary camel** | | |
| 7-16 months | Active weaning, majority 12-16 months (68%) | (20) |
| 7-18 months, average 11 months |  | (21) |
| 7-19 months, average 11 months | Longer lactation after birth in the dry season (winter and summer), cessation of lactation 2-4 months after new conception | (22) |
| 8-24 months | Active weaning | (23) |
| 10 months | If conception is within the first 30 days after parturition | (24) |
| 10-15 months | Active weaning in 45% of households | (25) |
| 12 months | Prolonged up to 3 years if mating is prevented | (26) |
| 12 months | Active weaning | (27) |
| 12-24 months, average 16 months | cessation of lactation 2-4 months after new conception | (28) |
| 14 months | cessation of lactation 2-4 months after new conception | (29) |
| 14-16 months | Pregnant females | (24) |
| Beyond 16 months | Non-pregnant females | (24) |
| 22 months | Non-pregnant females | (29) |

**S1 Text. Vicugna vicugna.** Additional publications on breeding seasonality of vicunas not included in Table 1

Besides the publications found in Table 2, the following studies mentioned the reproduction seasonality of vicunas. In Peru, births took place from February to April (7, 11, 30). Bosch and Svendsen (31) gave a breeding season from March to May. Franklin (3) mentioned that 75% of all births occurred during March, therefore most of the mating happened in April. Mating itself was observed by Menard (32) in March and April. Further South, in the Abra Pampa Experimental station in Argentina, calving was observed during the months of February to April (33). Another study at this experimental station described the reproductive season of vicuna from January to April (34). At the Los Andes Reserve in Argentina, reproductive season is described to fall together with the wet season, which is from December to March (35). All these publications hence indicate mating and birth mainly during austral autumn (short-day season).

**S2 Text. Vicugna pacos.** Additional publications on breeding seasonality of alpacas not included in Table 2

Besides the publications found in Table 3, the breeding seasonality of alpaca (*Vicugna pacos*) was mentioned in the following studies. In an experimental setting in New Zealand, Pollard et al. (36) reported that mating of alpacas was possible both in austral spring (mid-October to early December) and autumn (mid-February to March) when one male alpaca was introduced to a single female. During austral spring, females showed more dismissive behavior and males less sexual interest than in austral autumn. Under traditional management, the mating season was from late December to March (austral summer to autumn) (36). Other authors mentioned the mating season of alpacas in New Zealand from October to April (austral spring to autumn) (37, 38). In Australia, pregnancies were observed throughout the year, however mating was most successful in austral summer (39). In Peru, mating season was reported from January to April (austral summer and autumn) (40, 41) and January to March (austral summer and autumn) (42). Matching the observations from Pollard et al. (36) in New Zealand, Fernández-Baca et al. (43) noted that a seasonal mating of alpacas in Peru only occurred when female and male alpacas were housed together continuously all year round. Contrasting, reproduction was possible throughout the year if males were kept separately and were introduced to a group of females only once a month (43). On the Northern Hemisphere in Germany, alpacas were born from April to June (spring, beginning of summer) (44). In conclusion, most of these publications indicate mating and birth during austral autumn and some of them in austral summer. Further, some indications are present that mating is possible all year round when female and male alpacas are housed separately and only brought together for mating purposes.

**S3 Text. Lama guanicoe.** Additional publications on breeding seasonality of guanacos not included in Table 3

Besides the publications found in Table 4, the breeding seasonality of guanacos was mentioned in the following publications. An early mention by Dennler de la Tour (45) gave a mating season from November to February (austral summer) for guanacos in South America, without specifying the exact localization. Similarly, mating was observed to peak in December and January and lasted until February in Argentina (austral summer) (46). In the Andes of Chile, most of the births occurred between November and February (austral summer) but the first newborns were already seen in August (austral winter) (unpublished data by B. Gonzáles, cit. in Gonzáles et al. (47)). By contrast, Franklin (48) suggested a birth season during April, May and June (austral autumn) for nearly 60 percent of the females observed in Peru. Unpublished data by B. Gonzáles, cited by Gonzáles (47), observed neonates throughout the whole year at the coast of northern Chile, although they were more pronounced during the months of July to December (long-day period). These publications, hence, appear to cover the whole year, indicating seasonal reproduction but no common pattern.

**S4 Text. Lama glama.** Additional publications on breeding seasonality of llamas not included in Table 4

The following publications mentioned the breeding seasonality of llamas but were not included in Table 5. In Bolivia, the mating season is reported to happen from November until February (austral summer) which coincides with the rainy season (49). In the Peruvian highlands, mating season was mentioned from January to March (austral summer) (42). This statement is consistent with the results from an experimental setting in the months of February to April at La Raya research station in Peru. 96% of females ovulated two days after mating with an intact or vasectomized male in these months, therefore mating should be possible during the months of February to April at a minimum (austral autumn) (50). In an experimental setting in Argentina (Balcarce Experimental Station, southeast of Buenos Aires) the overall quality of semen was better in austral summer (experiments in February and March) than in austral spring (October and November) (51). Somewhat contradictory results were obtained by Giuliano et al. (52). They examined ejaculates of male llamas at the School of Veterinary Sciences in Buenos Aires, Argentina. Sperm concentration was greater and sperm tail abnormalities sparser in the austral winter collections (July to September) than in samples from collections in austral summer (February to April) (52). Most of these publications hence indicate mating and birth mainly during austral autumn, except for the last cited authors who reported better sperm quality for the austral winter months.

**S5 Text. Camelus bactrianus**. Additional publications on breeding seasonality of Bactrian camels not included in Table 5.

Very few studies report on the breeding seasonality of Bactrian camels (*Camelus bactrianus*). In Mongolia, Yadamsuren et al.(53) indicate a mating season for Bactrian camels from November to March (winter/spring). In another publication, Bactrian camels in Mongolia were observed from December to February (winter) for rutting behaviour. Unfortunately, the authors gave no information if mating was observed during this whole time (54). Other authors stated that the mating season of Mongolian Bactrian camels begins with the winter season, without specifying the exact months (55). Winter season was set to the months of December, January and February in Mongolia in another publication (56). In India, *C. bactrianus* showed seasonal reproduction in winter, extending from December to February or March (57). Rutting behaviour of males was observed in winter, without giving the location (58). The same authors also gave three birthdates of Bactrian camels (March and April), which corresponds to mating in February and March (58).

**S6 Text. Camelus dromedarius.** Additional publications on breeding seasonality of dromedary camels not included in Table 6

Various publications gave a breeding season for dromedary camels without providing detailed data. In Marocco, some authors mentioned a rutting season from mid-December to March (winter/ beginning of spring) (59), others gave a breeding season from October to April (autumn to spring) (60). In Israel, the breeding season extended from December to early April (winter/spring) (61) and in Oman, peak breeding season is mentioned from December to March (winter/ beginning of spring) (62). In Nigeria, calving and mating took place in the months of October to December (autumn/ beginning of winter) (63) and in another study, ovaries from slaughtered females were more active during November and December (beginning of winter) than during September and October (the remaining months of the year were not included in the study) (64).

For Saudia Arabia, the breeding season is described from autumn to spring (November to April (65, 66) or November to March (67)). November to March was also the breeding season noted by Skidmore et al. (68) for female dromedary camels on the Arabian peninsula. Other authors gave a mating season for males from November to July in Saudi Arabia (end of autumn to summer), but also mentioned that they believe that a male is capable of fertilizing a female in oestrus throughout the year (69). In agreement to this statement, Almutairi et al. (70) recorded births of dromedary camels in both seasons from October to February and March to September in Saudi Arabia. Nevertheless, they mention that the mating season started in September (autumn). Exact data of the birth records was not provided in this study (70). In the United Arabian Emirates, per-cycle pregnancy rates were examined between September and May. They found a non-significant increase in the months of November to February (winter) with highest per-cycle pregnancy rate in December (58.1%). In May when the study ended, the per-cycle pregnancy rate was still more than 40% and also at the start of the study in September, per-cycle pregnancy rate was already above 30%. No information is given for the remaining months of the year (71).

Other authors provided information on the breeding season based on testicular or ovarian characteristics or hormone measurements. In Egypt, a higher testicular sperm reserve was noted from December to May (winter/ spring) compared to the rest of the year (72). In another study, the maximum weight of testicles was recorded in March and the lowest in summer (given as June and July) (73). Testicular activity was also examined in Algeria. From November to February (winter) the volume of seminiferous tubules was increased and the average number of spermatozoa per seminiferous tubule was the highest. Although a spermatogenetic activity was noted throughout the year, it was highest during the rutting season (74).

In females, examination of ovaries of slaughtered animals in Algeria revealed less active ovaries in summer (July to October) than in winter (November to February) and spring (March to June), and no dominant follicles were observed in summer; the number of Corpora lutea was highest in winter (75). In Pakistan, higher levels of serum estradiol, T3 and T4 were found in non-pregnant female camels from November to April (winter/ spring) compared to the months of May to October. In the same study, serum progesterone and cortisol was lower from November to April than from May to October (76).

**S7 Text. Studies on photosensitivity**

In male dromedaries, testosterone concentrations, testicular volume, libido and mating ability scores were significantly higher four weeks after receiving a melatonin implant in the off-breeding season (duration of experiment: June to August; summer) compared to the control group without implants. Reproductive performance was improved in the treatment group in the following breeding season (December to February; winter) (77). Blindfolding of male dromedary camels for around three hours per day led to higher reproductive parameters such as testicular volume, libido and serum melatonin concentrations during the non-breeding season (June to August; summer) compared to a control group. Most spermatic parameters of the treatment group were similar to the parameters measured during the breeding season (December to February; winter), however sperm abnormalities were higher (78). These results would normally be interpreted as indications for short-day breeding but could also be interpreted as a preparation for reproductive activity towards the end of the short-day period.

Similar observations were made in females. Female dromedaries receiving melatonin implants two months ahead of the breeding season (December to March; winter/spring) showed increased follicular growth within one week of the treatment and the first animals reached ovulatory size follicles three weeks after the melatonin implantation. In the control group, none of the females had follicles of ovulatory size (79). In another experiment starting in October (autumn, natural breeding season December to March), female dromedaries were blindfolded for 6 hours per day during daytime for 8 weeks, leading to a prolonged daily dark phase. Follicular growth was detectable 3 weeks after the start of the treatment and continued to ovulatory size follicles with successful ovulation when mated. The control group showed small follicles as well but 3 weeks later than the treatment group and without progression in growth (80). In conclusion, dromedary camels respond to melatonin and blindfolding with increased reproductive activities.

In another study, female dromedaries were housed under an artificial long-day photoperiod (16L:8D) for 41 days at the end of the natural breeding season (October to April; autumn to spring) (60). One month after the beginning of this artificial photoperiod, endogenous melatonin levels were measured. The treatment group had lower melatonin levels (significant at only one time point) and a significantly shorter melatonin peak than the control group housed under natural photoperiod (14L:10D). Following the artificial photoperiod, the treatment group was put under natural light again and received melatonin implants on May 31^st^. Follicular growth was achieved one month later, which was 3.5 months ahead of the breeding season. Further, plasma 17β-estradiol levels were increased and plasma prolactin levels were moderately but statistically significant suppressed compared to the control group (60).

For Bactrian camels no publications were found on the influence of daylight length and reproductive activity. However, it was shown that ovarian granulosa cells express melatonin receptors (81), and that estradiol levels increased after adding melatonin to an in vitro granulosa cell culture (82). Further, melatonin receptors were detected in pineal glands (in pinealocytes und glia cells) of Bactrian camels (83). The pineal is an essential part of the pathway regulating the photic and endogenous melatonin synthesis in mammals (reviewed e.g. in (84)).

Studies on melatonin secretion and reproductive hormone concentrations were sparse in South American camelids. Only for guanacos some publications were found. Correa et al. (85) observed higher levels of plasma 17β-estradiol, LH and FSH and lower levels of melatonin in summer (December) than in winter (July). In another study (86), plasma prolactin concentrations were significantly higher during short-days (10L:14D) than during long-days (16L:8D). Further, animals of the short-day group received melatonin implants every 23 days for 6 weeks. After 21 days, a significant decrease in prolactin levels was noted (86). Prolactin is known to stimulate lactogenesis and galactopoiesis as well as being involved in various brain functions including regulations of maternal behavior (review e.g. (87)).

**REFERENCES**

1. Bravo PW. Chapter 60 - Camelidae. In: Miller RE, Fowler ME, editors. Fowler's Zoo and Wild Animal Medicine, Volume 8. St. Louis: W.B. Saunders; 2015. p. 592-602.

2. Chen BX, Yuen ZX, editors. Reproductive pattern of the Bactrian camel. The camelid: an all-purpose animal Vol 1 Proceedings of the Khartoum Workshop on Camels, 18-20 December 1979; 1984; Khartoum, Sudan: Scandinavian Institute of African Studies, Uppsala.

3. Franklin WL. Biology, ecology, and relationship to man of the South American camelids. Mammalian Biology in South America. 1982;6:457-89.

4. Riveros JL, Urquieta B, Bonacic C, Hoffmann B, Bas F, Schuler G. Endocrine changes during pregnancy, parturition and post-partum in guanacos (*Lama guanicoe*). Animal Reproduction Science. 2009;116(3):318-25.

5. San-Martin M, Copaira M, Zuniga J, Rodreguez R, Bustinza G, Acosta L. Aspects of reproduction in the alpaca. Reproduction. 1968;16(3):395-9.

6. Condori Rojas N. Estimación de parámetros genéticos para caracteres reproductivos en alpacas Huacaya (*Vicugna pacos*) del CIP Quimsachata del INIA-Puno: Universidad Nacional Jorge Basadre Grohmann-Tacna; 2014.

7. Franklin WL. Contrasting socioecologies of South America’s wild camelids: the vicuña and the guanaco. Advances in the study of mammalian behavior. 1983;7:573-629.

8. Koford CB. The Vicuna and the Puna. Ecological Monographs. 1957;27(2):153-219.

9. Tibary A, Johnson LW, Pearson LK, Rodriguez JS. Chapter 25 - Lactation and Neonatal Care. In: Cebra C, Anderson DE, Tibary A, Van Saun RJ, Johnson LW, editors. Llama and Alpaca Care. St. Louis: W.B. Saunders; 2014. p. 286-97.

10. Mössler M, Aichner J, Müller A, Albert T, Wittek T. Concentrations of fat, protein, lactose, macro and trace minerals in alpaca colostrum and milk at different lactation stages. Animals. 2021;11(7):1955.

11. Bravo PW, Fowler ME. Chapter 34 - Order Artiodactyla, Family Camelidae (Guanaco, Vicuñas). In: Fowler ME, Cubas ZS, editors. Biology, medicine, and surgery of South American wild animals. 1st ed. Ames, Iowa: Iowa State University Press; 2001. p. 392-401.

12. Garay G, Franklin WL, Sarno R, Johnson W. Development of juvenile guanaco social behavior: first study on a wild population from the Chilean Patagonia. Revista Chilena de Historia Natural. 1995;68:429-38.

13. Sarno RJ, Franklin WL. Maternal expenditure in the polygynous and monomorphic guanaco: suckling behavior, reproductive effort, yearly variation, and influence on juvenile survival. Behavioral Ecology. 1999;10(1):41-7.

14. Riek A, Gerken M. Measurements of the bodyweight and other physical characteristics of 11 llamas (*Lama glama*) from birth to weaning. Veterinary Record. 2007;161(15):520-3.

15. Fowler ME. Medicine and surgery of South American camelids: llama, alpaca, vicuña, guanaco: Ames, Iowa State University Press; 1989.

16. Johnson LW. Llama Nutrition. Veterinary Clinics of North America: Food Animal Practice. 1994;10(2):187-201.

17. Yao H, Dou Z, Zhao Z, Liang X, Yue H, Ma W, et al. Transcriptome analysis of the Bactrian camel (*Camelus bactrianus*) reveals candidate genes affecting milk production traits. BMC Genomics. 2023;24(1):660.

18. Peters J, Driesch Avd. The two-humped camel (*Camelus bactrianus*): new light on its distribution, management and medical treatment in the past. Journal of Zoology. 1997;242(4):651-79.

19. Chapman M. Mongolia: Bactrian camels. World Animal Review. 1985;55:14-9.

20. Faraz A, Younas M, Waheed A, Tauqir N, Khan N, Nabeel M. Study of production parameters in extensive kept Marecha dromedary camel at desert Thal. Advances in Animal and Veterinary Sciences. 2021;9(4):576-80.

21. Chamekh L, Khorchani T, Dbara M, Hammadi M, Yahyaoui MH. Factors affecting milk yield and composition of Tunisian camels (*Camelus dromedarius*) over complete lactation. Tropical Animal Health and Production. 2020;52(6):3187-94.

22. Bekele T, Zeleke M, Baars RMT. Milk production performance of the one humped camel (*Camelus dromedarius*) under pastoral management in semi-arid eastern Ethiopia. Livestock Production Science. 2002;76(1):37-44.

23. Tadesse Y. Husbandry and breeding practices of dromedary camels among pastoral communities of Afar and Somali regional states, Ethiopia. Journal of Agriculture and Environment for International Development. 2014;108(2):167-89.

24. Mehta S, Sharma A, Bissa U, Singh S. Lactation persistency, yield and prediction models in Indian dromedary. Indian Journal of Animal Sciences. 2015;85:875.

25. Traoré B, Moula N, Toure A, Ouologuem B, Leroy P, Antoine-Moussiaux N. Characterisation of camel breeding practices in the Ansongo Region, Mali. Tropical Animal Health and Production. 2014;46(7):1303-12.

26. Kaufmann BA. Reproductive performance of camels (*Camelus dromedarius*) under pastoral management and its influence on herd development. Livestock Production Science. 2005;92(1):17-29.

27. Chibsa MB, Mummed YY, Kurtu MY, Leta MU. Defining weaning age of camel calves in Eastern Ethiopia. SpringerPlus. 2014;3(1):313.

28. Mehta S, Bissa U, Patil N, Pathak K. Importance of camel milk and production potential of dromedary breeds. Indian Journal of Animal Sciences. 2011;81(11):1173-7.

29. Nagy P, Faigl V, Reiczigel J, Juhasz J. Effect of pregnancy and embryonic mortality on milk production in dromedary camels (*Camelus dromedarius*). Journal of Dairy Science. 2015;98(2):975-86.

30. Menard N. Quelques aspects de la socioecologie de la vigogne lama vicugna. Revue d'écologie. 1982;36(1):15-35.

31. Bosch PC, Svendsen GE. Behavior of male and female vicuna (*Vicugna vicugna* Molina 1782) as it relates to reproductive effort. Journal of Mammalogy. 1987;68(2):425-9.

32. Menard N. Le régime alimentaire des vigognes (*Lama vicugna*) pendant une période de sécheresse. Mammalia. 1984;48(4):529-40.

33. Aller J, Alberio R, editors. Diagnóstico de gestación por medio de ultrasonografía en vicuñas (*Vicugna vicugna*) en la puna argentina. Manejo Sustenable de la Vicuña y el Guanaco; 1998; Santiago, Chile.

34. Vila BL, Cassini MH. Time allocation during the reproductive season in vicuñas. Ethology. 1994;97(3):226-35.

35. Mosca Torres ME, Puig S, Novillo A, Ovejero R. Vigilance behaviour of the year-round territorial vicuña (*Vicugna vicugna*) outside the breeding season: Influence of group size, social factors and distance to a water source. Behavioural Processes. 2015;113:163-71.

36. Pollard JC, Littlejohn RP, Moore GH. Seasonal and other factors affecting the sexual behaviour of alpacas. Animal Reproduction Science. 1995;37(3):349-56.

37. Knight TW, Ridland M, Scott I, Death AF, Wyeth TK. Foetal mortality at different stages of gestation in alpacas (*Lama pacos*) and the associated changes in progesterone concentrations. Animal Reproduction Science. 1995;40(1):89-97.

38. Davis GH, Dodds KG, Moore GH, Bruce GD. Seasonal effects on gestation length and birth weight in alpacas. Animal Reproduction Science. 1997;46(3):297-303.

39. Vaughan J, Macmillan K, Anderson G, D'Occhio M. Effects of mating behaviour and the ovarian follicular state of female alpacas on conception. Australian Veterinary Journal. 2003;81(1‐2):86-90.

40. Urviola S J, Leyva V V, Mamani N R, Pérez G U, Urviola G A. Comportamiento sexual y cópula de machos castrados en la inducción de la ovulación en alpacas. Revista de Investigaciones Veterinarias del Perú. 2022;33(5):e23792.

41. Bravo PW, Sumar J. Laparoscopic examination of the ovarian activity in alpacas. Animal Reproduction Science. 1989;21(3):271-81.

42. Huanca W, Ratto M, Santiani A, Cordero A, Huanca T, Salamanca L, editors. Embryo transfer in camelids: study of a reliable superovulatory treatment in llamas. South American camelids research; 2004.

43. Fernández-Baca S, Sumar J, Novoa C. Comportamiento sexual de la alpaca macho frente a la renovación de las hembras. Revista de Investigaciones Veterinarias del Peru. 1972;1(2):115-228.

44. Grund S, Vogel M, Mülling CKW. Morphometric evaluation of the growth of Alpacas (*Vicugna pacos*) from birth to 36 months of age. Small Ruminant Research. 2018;166:61-5.

45. Dennler de La Tour G. The Guanaco. Oryx. 1954;2(5):273-9.

46. Panebianco A, Gregorio PF, Ovejero R, Marozzi A, Leggieri LR, Taraborelli PA. Reproductive flexibility in South American camelids: first records of alternative mating tactics in wild guanacos (*Lama guanicoe*). Mastozoología neotropical. 2020;27(1):200-5.

47. González BA, Palma RE, Zapata B, Marín JC. Taxonomic and biogeographical status of guanaco *Lama guanicoe* (Artiodactyla, Camelidae). Mammal Review. 2006;36(2):157-78.

48. Franklin WL. Guanacos in Peru. Oryx. 1975;13(2):191-202.

49. Wurzinger M, Willam A, Delgado J, Nürnberg M, Zárate AV, Stemmer A, et al. Design of a village breeding programme for a llama population in the High Andes of Bolivia. Journal of Animal Breeding and Genetics. 2008;125(5):311-9.

50. Adams GP, Sumar J, Ginther OJ. Effects of lactational and reproductive status on ovarian follicular waves in llamas (*Lama glama*). Reproduction. 1990;90(2):535-45.

51. Ferré LB, Malik G, Aller JF, Alberio RH, Fresno C, Kjelland ME. Llama (*Lama glama*) semen collection via thermo-electric artificial vagina: Effect of seasonality and collection interval on ejaculate characteristics. Small Ruminant Research. 2015;133:140-7.

52. Giuliano S, Director A, Gambarotta M, Trasorras V, Miragaya M. Collection method, season and individual variation on seminal characteristics in the llama (*Lama glama*). Animal Reproduction Science. 2008;104(2):359-69.

53. Yadamsuren A, Dulamtseren E, Reading RP, editors. The conservation status and management of wild camels in Mongolia. Camels in Asia and North Africa - Interdisciplinary perspectives on their past and present significance; 2012: Verlag der Österreichischen Akademie der Wissenschaften.

54. Reading RP, Blumer ES, Mix H, Adiya J, editors. Wild Bactrian camel conservation. Ecosystem Research in the Arid Environments of Central Asia: Results, Challenges, and Perspectives; 2005; Ulaanbaatar, Mongolia.

55. Saipolda T, editor Mongolian camels. ICAR Technical Series -  Current Status of Genetic Resources, Recording and Production Systems in African, Asian and American Camelids; 2004; Sousse, Tunisia: ICAR, Via G. Tomassetti 3, 1/A, 00161 Rome, Italy.

56. Yadamsuren A, Odonkhuu D, Shaochuang L. The seasonal distribution of wild camels (*Camelus ferus*) in relation to changes of the environmental conditions in Mongolia. Open Journal of Ecology. 2019;9(8):293-314.

57. Vyas S, Bissa U. Current status and strategies for conservation of double hump camel (*Camelus dromedarius*) in Ladakh, India. Journal of Livestock Science. 2019;12:132-7.

58. Wemmer C, Murtaugh J. Olfactory aspects of rutting behavior in the Bactrian camel (*Camelus bactrianus ferus*). In: Müller-Schwarze D, Silverstein RM, editors. Chemical Signals: Vertebrates and Aquatic Invertebrates. Boston, MA: Springer US; 1980. p. 107-24.

59. Charnot Y. Le cycle testiculaire du dromadaire. Bulletin de la Société des Sciences Naturelles et Physiques du Maroc. 1964;44:37-45.

60. El Allali K, Sghiri A, Bouâouda H, Achaâban MR, Ouzir M, Bothorel B, et al. Effect of melatonin implants during the non-breeding season on the onset of ovarian activity and the plasma prolactin in dromedary camel. Frontiers in Veterinary Science. 2018;5(44).

61. Elias E, Bedrak E, Yagil R. Estradiol concentration in the serum of the one-humped camel (*Camelus dromedarius*) during the various reproductive stages. General and Comparative Endocrinology. 1984;56(2):258-64.

62. Manjunatha BM, Al-Bulushi S, Pratap N. Synchronisation of the follicular wave with GnRH and PGF2α analogue for a timed breeding programme in dromedary camels (*Camelus dromedarius*). Animal Reproduction Science. 2015;160:23-9.

63. Abdussamad A, Holtz W, Gauly M, Suleiman M, Bello M. Reproduction and breeding in dromedary camels: insights from pastoralists in some selected villages of the Nigeria-Niger corridor. Livestock Research for Rural Development. 2011;23(8).

64. Yahaya M, Takahashi J, Matsuoka S, Alaku O. Ovarian activity of dromedary (single humped) camel (*Camelus dromedarius*) in North-Eastern Nigeria. Asian-Australasian Journal of Animal Sciences. 1999;12(6):868-70.

65. Al-Qarawi AA, Abdel-Rahman HA, El-Belely MS, El-Mougy SA. Intratesticular morphometric, cellular and endocrine changes around the pubertal period in dromedary camels. The Veterinary Journal. 2001;162(3):241-9.

66. Ghoneim IM, Waheed MM, Adam MI, Al-Eknah MM. Relationship between the size of the dominant follicle, vaginal electrical resistance, serum concentrations of oestradiol and progesterone and sexual receptivity during the follicular phase of the dromedary camel (*Camelus dromedarius*). Animal Reproduction Science. 2015;154:63-7.

67. Swelum AA-A, Alowaimer AN. The efficacy of controlled internal drug release (CIDR) in synchronizing the follicular wave in dromedary camels (*Camelus dromedarius*) during the breeding season. Theriogenology. 2015;84(9):1542-8.

68. Skidmore JA, Starbuck GR, Lamming GE, Allen WR. Control of luteolysis in the one-humped camel (*Camelus dromedarius*). Reproduction. 1998;114(2):201-9.

69. Arthur GH, A/Rahim AT, Hindi ASA. 7. Reproduction and genital diseases of the camel. British Veterinary Journal. 1985;141(6):650-9.

70. Almutairi SE, Boujenane I, Musaad A, Awad-Acharari F. Non-genetic factors influencing reproductive traits and calving weight in Saudi camels. Tropical animal health and production. 2010;42(6):1087-92.

71. Nagy P, Juhasz J. Reproductive efficiency of a non-traditional milk producing animal: the dromedary camel. In: Nagy P, Huszenicza G, Juhasz J, editors. WBC/ICAR 2008 Satellite Meeting on Camelid Reproduction; Budapest, Hungary2008. p. 66-9.

72. El-Kon I, Heleil B, Mahmoud S. Effect of age and season on the testicular sperm reserve and testosterone profile in camel (*Camelus dromedarius*). Animal Reproduction. 2011;8(3/4):68-72.

73. Abdel-Raouf M, Owaida MM. Studies on reproduction in camels (*Camelus dromedarius*) IV. Gross changes in the morphology of the testis in relation to age and season. Assiut Veterinary Medical Journal. 1974;1(1.2):213-24.

74. Gherissi DE, Afri-Bouzebda F, Bouzebda Z, Lamraoui R. Testicular morphology and stereological evaluation of the seminiferous tubules around the rutting season of sahraoui dromedary camel. Global Veterinaria. 2016;17(6):568-76.

75. Gherissi DE, Afri-Bouzebda F, Bouzebda Z, Bonnet X. Are female camels capital breeders? Influence of seasons, age, and body condition on reproduction in an extremely arid region. Mammalian Biology. 2018;93(1):124-34.

76. Ali S, Ahmad N, Akhtar N, Ahmad M. Hormonal profiles in the serum and follicular fluid of female camel (*Camelus dromedarius*) during the peak and the low breeding season. Pakistan Veterinary Journal. 2011;31(4):331-5.

77. Swelum AA-A, Saadeldin IM, Ba-Awadh H, Alowaimer AN. Effects of melatonin implants on the reproductive performance and endocrine function of camel (*Camelus dromedarius*) bulls during the non-breeding and subsequent breeding seasons. Theriogenology. 2018;119:18-27.

78. Swelum AA-A, Saadeldin IM, Ba-Awadh H, Alowaimer AN. Shortened daily photoperiod during the non-breeding season can improve the reproductive performance of camel bulls (*Camelus dromedarius*). Animal Reproduction Science. 2018;195:334-44.

79. Dholpuria S, Vyas S, Purohit GN, Pathak KML. Sonographic monitoring of early follicle growth induced by melatonin implants in camels and the subsequent fertility. Journal of Ultrasound. 2012;15(2):135-41.

80. Vyas S, Singh R, Purohit GN, Pareek P, Sahani M. Ultrasound evaluation of ovarian response to photoperiodic control measures in *Camelus dromedarius*. Veterinarski Arhiv. 2008;78(1):39-48.

81. Zhao SQ, Gao Y, Zhang Y, Yang XP, Yang Z. cAMP/PKA/CREB signaling pathway-mediated effects of melatonin receptor genes on clock gene expression in Bactrian camel ovarian granulosa cells. Domestic Animal Endocrinology. 2021;76:106609.

82. Zhao S-Q, Zhang Y, Gao Y, Yang X-P, Yang Z, Yang Z-J. The in vitro effects of melatonin and Cry gene on the secretion of estradiol from camel ovarian granulosa cells. Domestic Animal Endocrinology. 2021;74:106497.

83. Junjie H, Weihua C, Xiaoyu Z, Yong Z, Xingxu Z, Fadi L. The melatonin receptor in the bactrian camel pineal: cloning experiments and distribution studies. Journal of Camel Practice and Research. 2015;22(1):49-54.

84. ViviD D, Bentley GE. Seasonal reproduction in vertebrates: Melatonin synthesis, binding, and functionality using Tinbergen’s Four Questions. Molecules. 2018;23(3).

85. Correa LM, Moreno RD, Riveros JL. Hypothalamic-pituitary-gonadal axis response to photoperiod changes in female guanacos (*Lama guanicoe*). General and Comparative Endocrinology. 2024;347:114427.

86. Correa LM, Moreno RD, Riveros JL. The effect of photoperiod and melatonin on plasma prolactin concentrations in female guanaco (*Lama guanicoe*) in captivity. Reproduction in Domestic Animals. 2021;56(4):680-3.

87. Cabrera-Reyes EA, Limón-Morales O, Rivero-Segura NA, Camacho-Arroyo I, Cerbón M. Prolactin function and putative expression in the brain. Endocrine. 2017;57(2):199-213.
