# Supplementary material for: Breeding seasonality of Tylopoda: Expected patterns in Old World camelids but an exceptional pattern in South American camelids
Source: PLoS One. 2025 Dec 22;20(12):e0339382. doi: 10.1371/journal.pone.0339382 (PMC12721552; doi:10.1371/journal.pone.0339382)
Supplement: Attachment — Submitted filename: Clauss_CamelidSeaonsality_ReplyLetter_251121.docx [file pone.0339382.s004.docx]

Manuscript PONE-D-23-06724

Reply to Review

Dear Dr. Gisele Akemi Oda,

thank you for giving us the opportunity to revise our manuscript and respond to the referees' comments.

Please find attached the revised manuscript and supplement with track-changes marking all the changes; in the table below, new text is marked in grey shading. Our response to specific referee comments are given at the bottom of this letter.

We thank you for your time and hope the manuscript now meets the requirements of PLoS One.

Sincerely,

Nicole Pauli, Marco Roller, Marcus Clauss

See detailed responses to reviewers down below

***************************************************************************

**Editor:**

Copyrighted imagery: we had obtained permission from the toy manufacturer a decade ago and used these images a lot. But to avoid problems, we re-drew the icons and now present a version of the figure for which we hold the copyright ourselves.

Caption for Figure 6 provided.

Supporting information appropriately labelled.

***************************************************************************

**Referee 1:**

This is a thorough review of breeding seasonality in camelids. Data collection appears exhaustive and the evaluation and interpretation of the published data careful.

| Reviewer's comment (lines refer to the original submission) | Our response | Changes made in the text (lines refer to the revised version) |
| --- | --- | --- |
| However, I found it hard to follow the authors’ text, because they do not define the seasons in the Method section and in the tables always refer to increasing or decreasing daylength, not seasons.  In the text to the various species we are then told that breeding occurs mainly in autumn or spring. From the Tables I take it that autumn refers mainly to March, April and May in the South and Aug, Sep, and Oct in the North (??). This needs to be made explicit for all seasons in the Method section. Also, clearly define what are long or short days and how this relates to increasing or decreasing daylength. | Thank you for this input. We included a definition of short-day and long-day breeders (in the introduction, L64). In the methods, a definition of the season regarding the months was added (L151).  The reference to increasing or decreasing day-length in the tables was made, as it is more precisely regarding long-day or short-day breeding than the seasons. However, the seasons are better understandable for the reader, that’s why in the text we focused on seasons.  We looked through all mentions of seasons and months again and added the label austral wherever missing if the season is referring to the Southern Hemisphere; and if only months were mentioned in the original manuscript, we added the season. | L64ff: If animals mate within the period of increasing daylength, they are categorized as long-day breeders; animals mating during the period of decreasing daylength are called short-day breeders [1].  L154: The annual seasons were defined for the Northern Hemisphere as spring (March-May), summer (June-August), autumn (September-November), and winter (December-February). For the Southern Hemisphere, spring is September to November, and the subsequent seasons follow in the same 3-month-sections as in the Northern Hemisphere. Whenever a season refers to the Southern Hemisphere, it was labelled “austral”.  Austral/precision of season: changes made throughout the manuscript and the supplements |

| Also, I consider the reasoning (for example line 82) that species with a MIBL >12 months can be expected to be less seasonal breeders a bit too one-dimensional. If the ecology is sufficiently predictably bad or excellent (for mother or offspring) at certain seasons they will be forced into seasonal breeding no matter what the MIBL. | Thank you for this comment. In this part, we refer to an existing theory from the source we cite. We made this clearer now by specifically referring to ‘this author’. And in this section of the manuscript we’re simply arguing, that species with a MIBL >12months are expected to be less seasonal because of the high cost accompanied with a strict breeding seasonality, and greater costs are something to avoid in an evolutional point of view.  To our knowledge, the theory of Kiltie we explain here has not been refuted in a publication. But we followed the reviewer and added a sentence that ecology can overrule these ‘costs’. | Change made L. 71-84: The period between the birth of one litter and the conception of the next plus the gestation time was defined by Kiltie [2] as the “minimum possible interval between litters (MIBL)”. This author explained that highly seasonal species with a MIBL of more than 12 but less than 24 months will lose reproductive opportunity if there are only few favourable months for conception, as there would be a delay into the next year until mating could occur again after a birth. The opposite applies to seasonal species with a MIBL equal to or just less than 12 months (or a whole multiple of one year): even under highly seasonal breeding, offspring can be produced basically each year or at a regular cycle without lost periods [2]. To stay within this limit, there is most likely a strong adaptive value to shorten gestation periods in highly seasonal species [3]. Species with a short MIBL can thus afford to be seasonal breeders without losing reproductive opportunity, whereas species with a MIBL greater than 12 months are expected to be less seasonal breeders, due to the cost of lost opportunity [2], unless the waiting time is systematically filled by lactation. Arguably, regardless of the theory presented by Kiltie [2], environmental conditions might nevertheless select for a seasonal physiology if neonates have a low chance of survival outside of the favourable season. |
| --- | --- | --- |
| Some discussion of the phylogenetic relationship of these species might also help. It is to be expected that Vicuña and Alpaca and Guanaco and Llama, respectively, are more similar to each other in their response to photoperiod simply because of their ancestry. Are there any data on C. ferus (or C. bactrianus ferus as others classify it) from China or Mongolia? | Good point. We included a new section regarding the phylogeny of camelid species in the Introduction. | L110 ff: The phylogeny of camelid species is not yet fully understood and several hypotheses were proposed. The guanaco is most likely the ancestor of the llama [4, 5], but the situation for alpaca is more complex. Although indications have been found, that vicunas are the ancestors of alpaca [5], recent phylogenetic studies suggest that alpaca have derived in some part from both Lama and Vicugna clades and hybridisation played a role [4, 6]. In OWC, the dromedary camel, the Bactrian camel and the wild Camelus bactrianus ferus are thought to be three separate species, or subspecies, with an estimated divergence dating back to the Pleistocene, which excludes the wild Bactrian camel as a direct ancestor of the domestic Bactrian camel or dromedary camel [7]. |

| I found Fig. 1 not particularly helpful: The day length cycle between 10 and 17 hours is specific to a certain latitude and the selection of species shown appears rather arbitrary. | We added the term ‘schematic’ to make clear this is a scheme, and the examples are chosen to make both the predictions, and general reproductive biology, understandable. We added the term ‘hypothetical’ to photoperiod – to stress even more that this is a scheme, not a specific situation at a certain latitude. | L. 92: **Fig 1. Schematic associations between gestation length, breeding season, photoperiod and assumed birthing season in spring.** Schematic presentation of the associations between gestation length (arrows), the onset of the breeding season (dots at the beginning of the arrows), a hypothetical photoperiod (day length) and an assumed favourable birthing season in spring (dashed rectangle). Seasonal breeders with a short gestation length (e.g. Snowshoe hares *Lepus americanus*) are long-day breeders, mating and giving birth within the same season [8]. Seasonal ruminants with gestation lengths of less than a year are short-day breeders with a mating season in autumn [9]. Asian elephants with gestation lengths of nearly two years tend towards long-day breeding [10]. Camelids with a gestation length of around a year are therefore suspected to be long-day breeders – if they had a seasonal reproductive pattern. Graph modified from Hufenus et al. [10]; animal icons drawn by Jeanne Peter. |
| --- | --- | --- |
| L 35 here the ambiguity about definitions of day length surfaces: If both groups are expected to be long day breeders (which I interpret to be perhaps May to August in the Northern and November to February in the Southern hemisphere, then offspring would be borne either into long or decreasing daylengths again for SAC) or into autumn for OWC. Why should this lead to births in spring? | Long-day breeders = mating during the days with increasing daylength. We included a definition in the Introduction. | L64ff: If animals mate within the period of increasing daylength, they are categorized as long-day breeders; animals mating during the period of decreasing daylength are called short-day breeders [1]. |
| L 27, 53 adaption? Why not adaptation? | Was corrected to adaptation. | L22: Seasonal breeding is a common adaptation among mammals in seasonal environments, […]  L48: […] but the adaptation observed the most is seasonal breeding activity. |
| L 110 not necessarily long day but perhaps better “increasing daylength” breeders | See the definition of long-day breeder in the Introduction. | L64ff: If animals mate within the period of increasing daylength, they are categorized as long-day breeders; animals mating during the period of decreasing daylength are called short-day breeders [1]. |
| L 181 correct scientific name is Vicugna vicugna (correct also in Fig. 2) | Thank you, this was corrected.  Also corrected in the Supplement: S1 Table, S1 Text, S2 Text | Corrections made throughout text and supplement. |

1.	Lamming GE, Parkes AS. Marshall’s physiology of reproduction. Volume 1. Reproductive cycles of vertebrates: Churchill Livingstone; 1984. xvi + 842 pp. p.

2.	Kiltie R. Gestation as a constraint on the evolution of seasonal breeding in mammals. In: Boyce MS, editor. Evolution of life histories of mammals: theory and pattern: Yale University Press; 1988. p. 257-89.

3.	Clauss M, Zerbe P, Bingaman Lackey L, Codron D, Müller DWH. Basic considerations on seasonal breeding in mammals including their testing by comparing natural habitats and zoos. Mammalian Biology. 2021. doi: 10.1007/s42991-020-00078-y.

4.	Diaz-Maroto P, Rey-Iglesia A, Cartajena I, Núñez L, Westbury MV, Varas V, et al. Ancient DNA reveals the lost domestication history of South American camelids in Northern Chile and across the Andes. eLife. 2021;10:e63390. doi: 10.7554/eLife.63390.

5.	Kadwell M, Fernandez M, Stanley HF, Baldi R, Wheeler JC, Rosadio R, et al. Genetic analysis reveals the wild ancestors of the llama and the alpaca. Proceedings of the Royal Society of London Series B: Biological Sciences. 2001;268(1485):2575-84. doi: 10.1098/rspb.2001.1774.

6.	Wang Z, Li J, Gu Z, Chen C, Wang J, Guo Z, et al. Hybrid Origin of Alpaca (Vicugna pacos) During Domestication of Camelids in the Mid-Holocene. Molecular Ecology. 2025;n/a(n/a):e70167. doi: <https://doi.org/10.1111/mec.70167>.

7.	Burger PA. The history of Old World camelids in the light of molecular genetics. Tropical Animal Health and Production. 2016;48(5):905-13. doi: 10.1007/s11250-016-1032-7.

8.	Bookhout TA. Breeding biology of Snowshoe Hares in Michigan's upper peninsula. The Journal of Wildlife Management. 1965;29(2):296-303. doi: 10.2307/3798434.

9.	Zerbe P, Clauss M, Codron D, Bingaman Lackey L, Rensch E, Streich JW, et al. Reproductive seasonality in captive wild ruminants: implications for biogeographical adaptation, photoperiodic control, and life history. Biol Rev Camb Philos Soc. 2012;87(4):965-90. Epub 2012/07/10. doi: 10.1111/j.1469-185X.2012.00238.x. PubMed PMID: 22780447.

10.	Hufenus R, Schiffmann C, Hatt J-M, Müller DWH, Lackey LB, Clauss M, et al. Seasonality of reproduction in Asian elephants *Elephas maximus* and African elephants *Loxodonta africana*: underlying photoperiodic cueing? Mammal Review. 2018;48(4):261-76. doi: 10.1111/mam.12133.
